# Co‐designing a community lifestyle intervention program to reduce postpartum weight retention

**DOI:** 10.1111/hex.13905

**Published:** 2023-11-03

**Authors:** Maureen Makama, Helen Skouteris, Lisa J. Moran, Briony Hill, Leanne M. Redman, Siew Lim

**Affiliations:** ^1^ Monash Centre for Health Research and Implementation Monash University Clayton Victoria Australia; ^2^ Health and Social Care Unit, School of Public Health and Preventive Medicine Monash University Melbourne Victoria Australia; ^3^ Warwick Business School Warwick University Coventry UK; ^4^ Pennington Biomedical Research Center Louisiana State University Baton Rouge Louisiana USA; ^5^ Health Systems and Equity, Eastern Health Clinical School Monash University Box Hill Victoria Australia

**Keywords:** community, mothers' groups, participatory design methodology, postpartum lifestyle intervention, program components

## Abstract

**Background:**

Postpartum weight retention is a major contributor to obesity in later life resulting in long‐term health consequences in women. Postpartum lifestyle interventions are known to be effective in reducing postpartum weight retention and improving the overall health and wellbeing of mothers but have poor reach and engagement. This study describes the engagement of mothers with young children in the development of a theory‐ and evidence‐based intervention to reduce postpartum weight retention.

**Methods:**

A participatory design methodology with input from a community mothers' group, literature reviews and an expert advisory group was applied. Mothers who were members of ‘Mothers of Preschoolers’ (MOPS) were invited to participate in a focus group discussion and two co‐design workshop sessions.

**Results:**

Thirteen women participated in a focus group discussion and 12 women in each co‐design workshop. We found that mothers valued having social support from their peers, practical support such as meal delivery, and learning opportunities that focus on the mother's health and wellbeing. The advisory group suggested leveraging the unique skills and prior experiences of mothers within the group and developing a curriculum that mothers can be trained to deliver.

**Conclusion:**

A program that emphasizes the strengths and value of mothers can increase their self‐worth and self‐confidence resulting in intrinsic motivation to improve lifestyle behaviours. An intervention designed to be implemented by MOPS for its members and incorporated into their regular sessions has the potential for feasibility and acceptability among mothers with young children.

**Patient or Public Contribution:**

Mothers with young children were part of the program planners and were involved in the design and conduct of this study and in the interpretation of the findings. A member of a community mothers' group recruited other mothers with young children within the group to participate in a series of sessions to discuss their experiences of the postpartum period and preferences for a lifestyle program. The mothers identified the behavioural outcomes and program goals for a postpartum lifestyle program and then generated the program ideas based on these.

## 1 INTRODUCTION

Obesity is a global epidemic affecting millions of people worldwide.^1^ According to the World Health Organization, in 2016, 40% of women aged 18 years and over were living with overweight, and 15% with obesity globally.^1^ Childbearing is associated with a substantial increase in weight and abdominal adiposity in women of reproductive age.^2^ Postpartum weight retention contributes significantly to this with more than 20% of women retaining >4 kg by 1 year postpartum.^3^, ^4^, ^5^, ^6^ Postpartum weight retention is associated with poorer outcomes in subsequent pregnancies and adverse maternal outcomes such as cardiovascular diseases, type 2 diabetes, metabolic syndrome, and long‐term maternal obesity in later life.^7^, ^8^, ^9^

Evidence on the efficacy of lifestyle interventions that target diet and physical activity in reducing postpartum weight retention is substantial.^6^, ^10^ However, interventions are typically characterized by poor uptake and engagement by postpartum women, reducing their impact at a population level.^10^ Competing demands on the mother's time in the postpartum period may lead to a lack of prioritization of healthy lifestyle behaviours and returning to a healthy weight. Hence, postpartum women can be hard to reach and engage in lifestyle interventions.^6^, ^11^ There is a clear imperative for implementation strategies to address barriers to intervention uptake and engagement. Such strategies are hypothesized to foster the effective translation of evidence on the efficacy of postpartum lifestyle interventions into practice in real‐world settings.^6^

One strategy to address barriers to engagement is to co‐design lifestyle intervention programs with mothers with young children as part of the planning, development, and implementation processes.^6^ Meaningful engagement of key stakeholders at each stage of intervention development is necessary to overcome practical obstacles to program implementation and improve the likelihood of positive health impacts.^12^ Community participatory approaches are effective in reaching hard‐to‐reach population groups and are key to driving improvement in healthcare by ensuring that contextual factors are captured in intervention development.^13^ As recommended by the UK Medical Research Council, it is also vital that intervention programs are grounded in theory and evidence to increase their effectiveness by targeting the determinants of behaviour and facilitating an understanding of what works.^12^, ^14^, ^15^, ^16^ Intervention mapping (IM) is a planning framework that provides a systematic process for effective step‐by‐step decision‐making for intervention planning, development, implementation and evaluation.^17^, ^18^ It has been extensively applied for the design of complex behaviour change interventions and has previously been combined with participatory design approaches for intervention program development.^17^, ^19^, ^20^

Health professionals have limited time and resources to provide ongoing lifestyle support to mothers after childbirth; therefore, integrating health promotion into existing services such as mothers' groups is both innovative and practical to achieve ongoing support for postpartum women.^11^, ^21^ There are international and local groups that engage women during the postnatal period (e.g., Mothers of Preschoolers and Centre of Perinatal Excellence [COPE]), offering opportunities to embed curriculum specific to different topics of value to mothers.^22^, ^23^ Mothers of Preschoolers (MOPS) is an international organization, with groups across more than 70 countries, that partners with churches and organizations worldwide to support mothers.^22^ MOPS focuses on the mother's wellbeing and provides structured and organized childcare for mothers during MOPS sessions.^24^

The aim of this study was to describe the co‐design of the components of a postpartum lifestyle program with MOPS, a community mothers' group. This study addressed the research question: what intervention components are preferred by mothers with young children in a community mother's group to reduce postpartum weight retention and improve health and wellbeing?

## 2 METHODS

### 2.1 IM and co‐design processes

IM is an iterative six‐step process with each step consisting of several tasks, which once completed, informs the next step.^17^ The steps in IM are (1) needs assessment—logic model of the problem; (2) defining program outcomes and objectives—logic model of change; (3) program design; (4) program production; (5) program implementation plan; (6) program evaluation plan. The current study presents a modified IM approach that includes steps 1–3 as described below. Steps 4–6 were beyond the scope of this research.

### 2.2 Theoretical framework

This study is underpinned by two theoretical evidence‐based frameworks: the Theoretical Domains Framework (TDF) and the Capability, Opportunity, Motivation and Behaviour (COM‐B) model.^25^, ^26^, ^27^ The TDF is an integrative framework that can facilitate a comprehensive assessment of behavioural determinants.^26^, ^27^ The TDF consists of 14 domains that are an expansion of the three core components of the COM‐B model and together form the hub of the Behaviour Change Wheel, a method for characterizing and designing behaviour change interventions.^25^

### 2.3 Participants

The co‐design process focused on two levels of the socioecological model—individual (mothers with young children) and organizational (mothers' group—MOPS) levels. Mothers with young children (5 years and under) were recruited from MOPS, a community of mothers with preschoolers who meet together to be supported, encouraged and equipped as they raise children. This research was conducted in partnership with one MOPS group in Metropolitan Victoria Australia.

### 2.4 Step 1: Needs assessment

Step 1 involved an assessment of the determinants of behaviour and environmental contributors to the problem (postpartum weight retention) the program seeks to address through epidemiological, behavioural and social analysis of the population at risk (mothers with young children). A thorough assessment of the capacity and needs of the target population (mothers with young children) and the program setting (MOPS) was undertaken to inform the program goals.

#### 2.4.1 The program planning group

Program planning and advisory groups were established to provide input, guidance and oversight in the program component design process. The planning group included 12 mothers with young children who were members and/or leaders of MOPS and four researchers with expertise in postpartum lifestyle research, dietetics, behavioural sciences, implementation science and health psychology. The advisory group (*n* = 2) were researchers with PhDs who have expertise in health psychology, maternal health, lifestyle medicine and program component design.

#### 2.4.2 Literature reviews

The needs assessment included literature reviews and focus group discussions. The following reviews were conducted: (i) a systematic review and meta‐analysis to evaluate the intervention characteristics associated with weight loss in postpartum women using the Template for Intervention Description and Replication framework^28^; (ii) a systematic review and meta‐analysis to identify the most effective behavioural strategies in changing postpartum women's physical activity and healthy eating behaviours^29^; (iii) a narrative review to examine the implementation challenges of postpartum lifestyle interventions^6^; and (iv) a mixed‐methods systematic review to understand the barriers and facilitators to a healthy lifestyle in postpartum women from the perspective of women and healthcare providers mapped to the TDF and COM‐B domains.^11^

#### 2.4.3 Focus group discussion

A focus group discussion was conducted over Zoom (version 5.11.1) with mothers of children 5 years and under who were members of MOPS recruited by convenience sampling. Participants were approached via email, and their contacts were obtained through a MOPS leader who was also a member of a consumer group for cardiometabolic health research in postpartum women. The discussion guide (Supporting Information S1: Table S1) followed the strengths, weaknesses, opportunities and threats approach to understanding the capabilities of MOPS in supporting the health and wellbeing of its members.^30^ The discussion was facilitated by a female allied health professional (MSc.) conducting research with postpartum women (M. M.). Two female dietitians and researchers with PhDs (L. J. M. and S. L.) were also present and took notes during the discussion. The researchers had no prior relationship with the participants. Participants were aware that the study was part of the facilitator's PhD research. The discussion was video‐recorded and transcribed verbatim by a transcription service provider, *GoTranscript*. The transcribed data were thematically analysed using NVivo, the text was reviewed line‐by‐line and initial codes were assigned. These codes were then organized and refined to generate subthemes and themes through an iterative process by two authors (M. M. and S. L.) (Supporting Information S1: Table S2).^31^

### 2.5 Step 2: Program outcomes and objectives—The logic model of change

The second step focused on specifying detailed outcomes for the program. During a co‐design workshop with the planning group, behavioural outcomes at the individual (mothers with young children) level and environmental outcomes at the organizational level (MOPS) were specified. Drawing on information generated during step 1, performance objectives (explicit behaviours needed to achieve each behavioural and environmental outcome) were specified for each outcome. A matrix of change objectives (what needs to change in the identified determinants to achieve the performance objectives) was constructed by mapping performance objectives to the determinants of behaviour according to the TDF and COM‐B model. A separate matrix was created for individual and organizational levels.

### 2.6 Step 3: Program design

In this step, the planning group worked from the logic model of change established in step 2 to conceptualize and design the program components. Theoretical methods (i.e., behaviour change techniques [BCTs] for influencing determinants of the target population) known to be effective in achieving the change objectives^32^, ^33^ were identified and mapped to the determinants and change objectives by one researcher (M. M.). The identification of BCTs was conducted using published literature.^32^ The change objectives grouped under each determinant were then operationalised into practical strategies that can be implemented within MOPS. Practical program strategies (program ideas or specific activities) were generated by the planning group during a second co‐design workshop by workshopping tools, skills and processes needed to achieve the program outcomes and objectives. It was also informed by the findings of the needs assessment. The program ideas were discussed and ranked according to their feasibility, impact and acceptability. An expert advisory group was consulted to further refine the program components. Following the workshops, practical strategies aligned with the change methods were identified and integrated with the program ideas generated by the planning and advisory groups. The findings of the focus group discussion and co‐design workshops were shared with the participants for comments or correction. Participants did not request any changes.

## 3 RESULTS

### 3.1 Participant characteristics

Thirteen mothers who were members of MOPS participated in the focus group discussion and 12 in the co‐design workshops. One participant dropped out due to other commitments. The mean age of participants was 37.1 ± 4.8 years. More than two‐thirds of the participants (69%) were born overseas: Saudi Arabia (*n* = 1), Malaysia (*n* = 2), China (*n* = 2), Hong Kong (*n* = 1), Philippines (*n* = 1), and United States (*n* = 1). Only four (31%) were born in Australia. Most participants were university graduates, while one had an advanced diploma, and another one only completed high school. Only five participants (39%) were in paid employment. The number of children of participants ranged from one to four (mean 2.4).

### 3.2 Step 1: Needs assessment—The logic model of the problem

#### 3.2.1 Literature reviews

The findings of the literature reviews have all been published previously^6^, ^11^, ^28^, ^29^ and are summarized in Supporting Information S1: Table S3. Postpartum lifestyle programs that include diet and physical activity components, delivery by a health professional, and behavioural strategies relating to self‐regulation are effective for weight management.^28^, ^29^ Some gaps were identified in the implementation of postpartum lifestyle programs such as not including postpartum women and community members as key stakeholders in the development process and suboptimal reporting of intervention characteristics.^6^ Barriers and facilitators to engaging in healthy lifestyle behaviours in the postpartum period were identified, for example, lack of knowledge regarding the benefits of healthy lifestyle behaviours, social support, and limited time and skills of health professionals in providing lifestyle support.^11^ These barriers contribute to low engagement in postpartum lifestyle intervention programs.^6^

#### 3.2.2 Focus group

The findings of the focus group discussions provided insight into the barriers and facilitators faced by MOPS in supporting the health and wellbeing of its members (Table 1). Six themes emerged from the thematic analysis of the focus group discussions (i) creating a supportive environment that enables peer and social support; (ii) providing practical support such as delivering meals; (iii) inspiring leadership from MOPS leaders that values motherhood; (iv) providing learning opportunities that focus on mothers' health and wellbeing; (v) reach and accessibility of MOPS; (vi) lack of resources for childminding. MOPS sought to create a supportive environment to encourage relationship building between mothers and other members of the community and to support healthy family relationships. MOPS provides an avenue for mothers to enjoy the company of one another and be supported by their peers. MOPS wanted to provide inspiring leadership with an emphasis on pastoral care and championing mothers to make them feel loved and valued. MOPS sought to nurture mothers in a holistic way targeting the physical, mental, social, emotional and spiritual wellbeing of the mother. This could be strengthened through the inclusion of physical activity components within MOPS sessions and by creating learning opportunities through engaging expert speakers. They considered it important for them to provide practical support such as meal delivery or assistance with childminding, especially in the early postpartum period and to mothers experiencing adversity. MOPS felt a need to expand their reach and make programs more accessible to mothers and felt limited in resources to accommodate more mothers because they relied on volunteers for childminding.

**Table 1:** Facilitators and barriers faced by MOPS in improving the health of its members.

Themes	Subthemes	Quotes
Creating a supportive environment that enables peer and social support	Building relationships between mothers and others in the community	even just the health of my relationships with my husband and my children to realize that this stage that we're in, some of the things I'm encountering is normal. #4
		They're the grandmother figure, the grandfather figure, which is wonderful, especially in our community where there are a lot of migrant moms connecting in with the group, and they don't have extended family living locally. #12
	Supportive environment	They're encouraging you to bring your kids and you know, the picture that they have of them working out. Um, just has toddlers everywhere and I just go, ‘Oh, okay. Well, that makes it so much more welcoming because I know my two will climb all over me.’ Um, but so will everyone else's, and I think it just makes it more comfortable because you know, that that's accepted and it's okay. #13
		And so I keep coming back to a program such as this because I think it provides beautiful community encouragement and support from others. I think it's good for mothers to see that they're not going through the journey alone. #8
	Peer support	And, uh, is‐is very healthy environment for a mother. And I find out they open my mind and they respect me the way I am, and they connect me with the community straight away, and is changed me. #10
		Um, and so just hearing other women and other moms share about their struggles. I started to realize like, ‘Oh, I'm not the only one going through this particular struggle.’ #4
	Provides company	looking for like company and‐and other moms that are at home that can share their time with me. #5
		Like when I‐when I finally made it to MOPS, it was almost just like a‐a breath of fresh air just over me because, um, if I didn't, you know, I‐I missed the connection and it just feeds my soul. #7
Providing practical support such as delivering meals	Practical support	free meals are funded to reach out to bless those moms who, um, you know, who just got a recent diagnosis that their child is, um, experiencing something just so shocking. #12
		I think even just offering to bring over a meal and, um, a cup of tea or to look after children. #13 …our group is really good at doing is to provide meals for each other and during lockdown, we did, um, like food hug deliveries. #12
		So like if I need a babysitter, for instance, I could say, ‘Hey,’ at anybody, ‘Can I drop my kids…’ And then … I can reciprocate that, um, you know, take someone else's kids. Um, and so the support can continue, even outside of the actual meeting. #4
		when I had COVID, um, cause my family is far, all my friends are far … the MOPS community was the one who came and, you know, dropped off medication and just, yeah, just helped me because they were local. So that's been really, really helpful. #6
	Mothers' resort/hotel/restaurant	the other aspect of‐of having a built‐in restaurant is that moms who aren't staying in this hotel, um, they could order these nutritious‐these nutritious meals that have been specially curated, right, for the wellbeing and the nurturing of moms as they're breastfeeding their kids, or, um, whatever part of their pregnancy journey, um, they're on perhaps. #12
Inspiring leadership that values motherhood	Values motherhood	just the fact that … you know, that you're not alone. You know that you're not such a failure as a mom, you know, it just gave me so much confidence and I've only attended one session … I pulled myself out from, and now I'm‐I'm seriously eating better. And, you know, I just—it just gave me so much confidence and I'm eating better … I actually watch what I wear, you know, I actually like try to look good because, and feel good because, yeah, because I guess I was uplifted. #11
		sometimes you even lose a bit of yourself because you're like, ‘Where's my identity now?’ … I've left a career to become a mother, … so I just love how MOPS champions mothers. It gives opportunity for them to have their souls fed … And then the mother's are nurtured as well. #8
		MOPS is so intentional to sow into the mom's life and to build relationships between the moms and just there's so much joy. So I think that that is so good for our health. #8
		being introduced to MOPS by the actual CEO of MOPS International. She, like, flew down from America and, like, gave a talk about—Um, like, a really inspiring talk. #2
		love radically because MOPS was birthed from this desire to want to, um, not only cherish and nourish mothers, so that they may flourish. #2
	Mentoring from older mums	Um, from there, uh, we have mentors as well. So moms who have raised their children past the preschooler age that can speak into, um, and give hope and encouragement to moms who are still in‐in that, um, stage of parenting preschoolers. #12
	Pastoral care	I think too, that's also what makes MOPS such a unique place because you can freely talk about, um, your beliefs as well and‐and have that Christian support network too, um, or extend that pastoral care. #13
Provides learning opportunities that focus on mothers' health and well‐being	Focuses on mother's mental health and wellbeing	but also I would love to see education for, uh, the mental health as well. hard for us to admit that we might be struggling in one area or another, but, um, with two of my three children, I had—I'm pretty sure I was struggling with undiagnosed postpartum depression. #4
	Focus on physical health	That was the biggest thing. Um, and an understanding like how foods could give me an insulin spike and enable me to gain more weight. So, um, yeah, so physical health has always been in the forefront of my mind, but I've never‐I've never engaged in a community, uh, group to focus on that part of my wellbeing. #1
	Engage experts	local dentist or local pediatrician, um, experts, uh, we've had, uh, authors come in as well, um, who might share about, um, one really powerful session was about motherly guilt and um, yeah, so just thinking about the theme and then reaching out for community support. Sometimes also, if there are mothers within our group who have expertise or work experience in a particular field, we get them to share as well. #12
	Health education	We discuss different questions along that theme that we had for that day … group discussion time has been particularly enriching for me. #4
		what makes MOPS unique and special is that it's moms helping moms that there's a‐a limit to what moms can actually do, because you have your own kids who are pre‐schoolers. Um, and you may also be juggling work with all of that so you're really time for, in trying to give to others and‐and trying to extend it and grow it. #13
		The first and foremost thing would have to be education. Um, it's fair enough that, um, like we all need to eat to live, but choosing the right foods for us is something that has to be told to us by a professional. #1
		I think, too, adding to that pelvic floor education too. Um, that's something I've struggled with quite a bit and nobody told me. I didn't end up working on my pelvic floor until I almost got pregnant with my third child and, um, so I thought, you know, am I just broken. #4
Reach and accessibility of MOPS	Partnership with other organizations to promote the health of mothers	it's who can you get on board to help with childcare or to make those connections with other organizations, um, or to approach other, you know, to make those applications for grant funding and for all that type of thing. #3
	Expand reach and access	it was really good to have online as well, because sometimes you just—it doesn't fit your schedule, or, um, you just can't make it or the kids are sick … So yeah, I really enjoyed the online gatherings actually. #9
		people who are further out, it would be lovely to have those little communities spring up. #4
		… last year doing lockdown, MOPS was … online, um, and I could join because, um, you know, all my kids are asleep. And it was like, just to catch up with other mums and there was heaps of support from the MOPS group…. And, um, it was just really good for my mental health, because, um, it was really hard time. #9
	Time and place barriers	So I‐I would just love to see it expand and grow and be available, um, at more times than just what it is. #13
	Limited resources	o I think, um, you know, it's who can you get on board to help with childcare or to make those connections with other organizations, um, or to approach other, you know, to make those applications for grant funding and for all that type of thing. #13
		I think too, because it's also affiliated with a religious, um, organization that a lot of grants, um, you know, there's the local council grant, but we're not eligible for that because it's affiliated with the church. #13
Lack of resources for childminding service	Limited childminders	I talked to MOPS about a lot of friends that I come across in the community, but I realize, um, I have to be mindful of the capacity of the carers and‐and, you know, space wise because we don't want to, um, over saturate the‐the carers space with children. #7

Abbreviation: MOPS, Mothers of Preschoolers.

#### 3.2.3 Program goal and logic model

**Figure 1:**
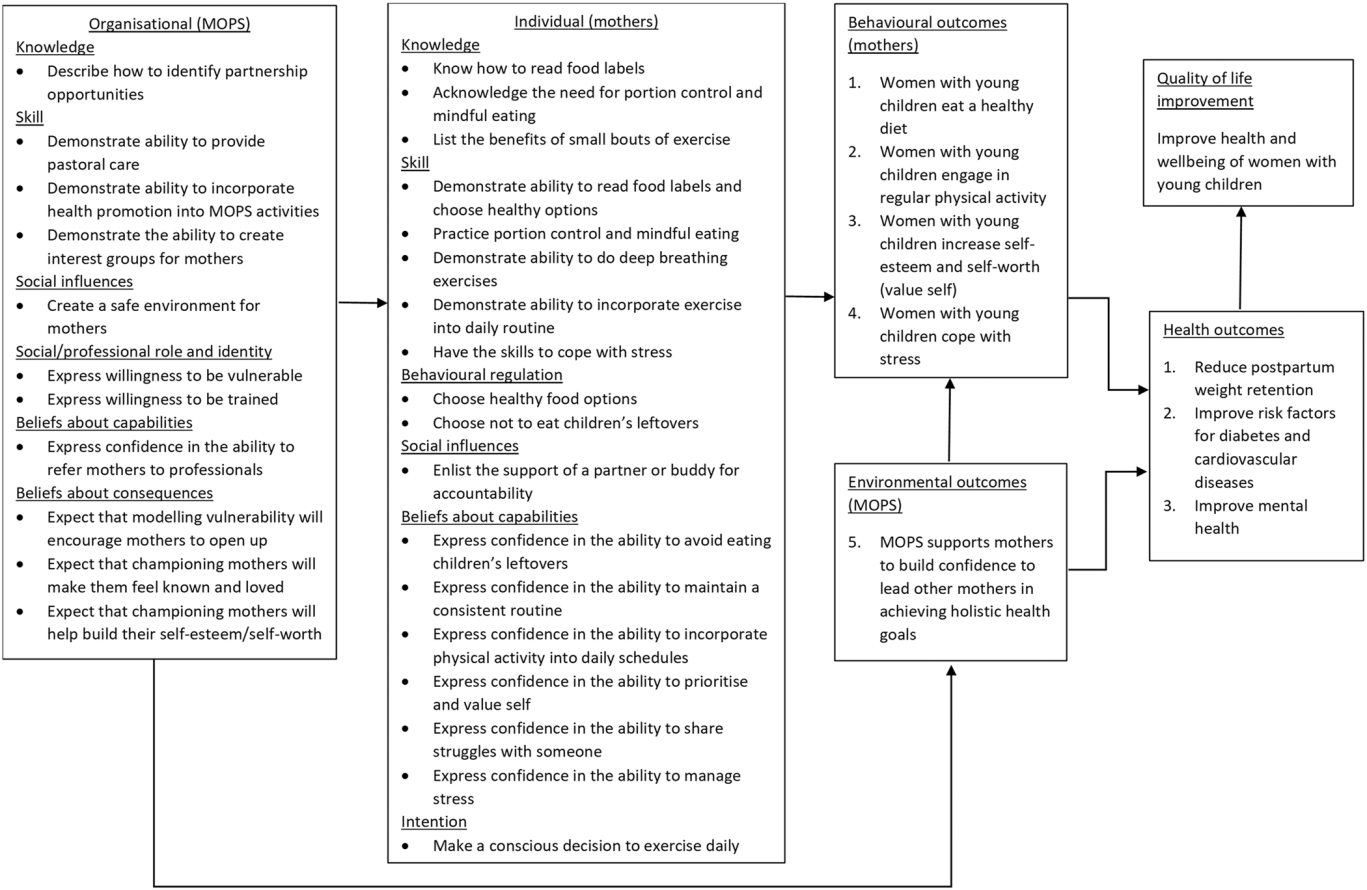
Logic model of MOPS. MOPS, Mothers of Preschoolers lifestyle program.

The findings of the needs assessment informed the development of a logic model identifying key areas to target and the program goals (Figure 1). The overall goal of the program was to reduce postpartum weight retention and improve the health and wellbeing of mothers with young children. We decided to focus on the behaviour of mothers with young children (individual level) as the target population group and the MOPS mothers' group (organizational level) as the environment for program implementation. Using the TDF and COM‐B as a guide, the logic model summarized the key determinants, behavioural outcomes and health outcomes for each potential level of the program (Figure 1).

### 3.3 Step 2: Program outcomes and objectives

Table 2 shows the program outcomes and specific performance objectives relating to each potential level of the program (mothers and MOPS). The behavioural outcomes chosen by mothers were to eat a healthy diet, engage in regular physical activity, increase self‐esteem and self‐worth and cope with stress. The outcome for MOPS as an organization was to support mothers to build confidence to achieve holistic health goals. Mothers' knowledge, skills, behavioural regulation, social influence, beliefs about capabilities and intentions were identified as determinants of mothers' behaviour at the individual level. At the organizational level, knowledge, skills, social influences, social/professional role and identity, beliefs about capabilities and beliefs about consequences of MOPS were identified. Using the information from step 1, the selected determinants (according to the TDF and COM‐B model) were mapped to the performance objectives to create change objectives for the program as shown in Supporting Information S1: Table S4.

**Table 2:** Program outcomes and performance objectives for the intervention by socioecological level according to mothers of young children and MOPS.

Program goal	Target group	Program outcome	PO
To improve the health and wellbeing of postpartum women	Postpartum women (individual)	Eat a healthy diet	*Mothers of young children will*: PO 1.1 Eat less sugar and refined carbohydrates and more healthy fats PO 1.2 Not eat children's leftovers PO 1.3 Be conscious of the food they eat
		Engage in regular physical activity	*Mothers of young children will*: PO 2.1 Have a consistent routine for newborn and self PO 2.2 Use deep breathing exercises PO 2.3 Understand that even small amounts of exercise each day is okay, for example, 7 min a day, 1 min per time PO 2.4 Incorporate exercise into usual routine, for example, house cleaning, playdates PO 2.5 Have someone to be accountable to, for example, partner or buddy
		Increase self‐esteem and self‐worth (value self)	*Mothers of young children will*: PO 3.1 Value and prioritize themselves PO 3.2 Be willing to share struggles with someone
		Cope with stress	*Mothers of young children will*: PO 4.1 Learn strategies to cope with stress, for example, allocate a 1‐h stress time per day
	MOPS (organizational)	Support mothers to build confidence to lead other mothers in achieving holistic health goals	*MOPS will*: PO 5.1 Partner with local health/allied health professionals to provide education/training opportunities to mothers PO 5.2 Refer mothers to professionals for help as needed PO 5.3 Create a safe space for mothers to be vulnerable PO 5.4 MOPS leaders model vulnerability as a virtue PO 5.5 Be intentional about pastoral care PO 5.6 Be intentional in championing mothers, serving them in the way they need and making them feel known and loved PO 5.7 Incorporate exercise, dance and music, healthy eating, and health promotion into MOPS structure/activities/discussions PO 5.8 Create interest groups for mothers, for example, meal planning, diet, weight loss PO 5.9 MOPS leaders take up training opportunities to equip them to better support mothers, for example, by PANDA

*Note*: Program outcomes and performance objectives were derived from the co‐design process.

Abbreviations: MOPS, Mothers of Preschoolers; PANDA, Perinatal Anxiety and Depression Australia; PO, performance objectives.

### 3.4 Step 3: Program design

The theoretical methods and practical strategies for achieving the change objectives are shown in Table 3. Some program ideas generated by the planning group include having expert speakers, for example, dietitians and psychologists; peer coaching through interest groups; incorporating a physical activity component into MOPS sessions; having one or two mentors for the group; shared exercise together, for example, walking groups; speed sharing session; recipe cards in a shared/combined meal; buddy system (pairing); cooking demonstration; book sharing in a discussion thread; and health goal setting with a buddy. These program ideas (practical strategies) were aligned with the behaviour change methods and formed the components of a potential intervention program (Table 3). For example, for the change objective relating to demonstrating an ability to incorporate exercise into daily routine, the theoretical methods deemed potentially effective were goal setting and self‐monitoring of behaviour. Practical strategies such as health goal setting with a buddy, walking groups, including physical activity components in MOPS sessions, and journaling were considered useful in delivering the method for mothers in MOPS.

**Table 3:** Example of strategies to achieve change objectives for mothers (individual level) and MOPS (organizational level).

Level of intervention	Determinants of behaviour	Change objectives^a^	Methods (BCTs)^b^	Practical strategies^c^
Mothers with young children (individual)	Knowledge	K1.1, K2.3	Instruction on how to perform the behaviour 4.1	Expert speaker in MOPS sessions
		K1.1, K2.3	Information about antecedents 4.2	Group discussions
	Skills	S1.1	Instruction on how to perform the behaviour 4.1 Demonstration of the behaviour 6.1	Cooking demonstration
				Expert speaker in MOPS sessions
				Group discussion
		S1.1, S2.4	Self‐monitoring of behaviour 2.3	Journaling
		S1.2, S1.3, S2.4	Goal setting (behaviour) 1.1	Health goal setting with a buddy
				Walking groups
				Physical activity component in MOPS sessions
		S1.2, S1.3	Self‐monitoring of behaviour 2.3 Habit reversal 8.4	Health goal setting with a buddy
		S2.2	Skills training 4.1	Guided practice
				Demonstration in MOPS sessions
		S2.2	Instruction on how to perform the behaviour 4.1 Demonstration of the behaviour 6.1	Guided practice
		S4.1	Self‐affirmation 13.4	Sharing sessions/speed dating in MOPS sessions
	Behavioural regulation	BR1.1, BR1.2	Self‐monitoring of behaviour 2.3	Health goal setting with a buddy
		BR1.1	Feedback on behaviour 2.2	Health goal setting with a buddy
		BR1.1, BR1.2	Action planning 1.4	Health goal setting with a buddy
	Social influences	SI2.5	Mobilizing social support (practical) 3.2	Buddy system (pairing)
			Modelling (positive role models, reinforced models) 6.1	Buddy system (pairing)
			Social reinforcement 10.5	Buddy system (pairing)
	Beliefs about capabilities (self‐efficacy)	BC1.2	Self‐monitoring of behaviour 2.3	Health goal setting with a buddy
		BC2.1	Goal setting 1.1	Goal setting, observation, reflection
		BC2.4	Commitment 1.9	Goal setting, observation, reflection
		BC3.1	Self‐affirmation 13.4	Sharing sessions/speed dating in MOPS sessions
		BC3.2	Self‐talk (positive self‐talk) 15.4	Sharing sessions/speed dating in MOPS sessions
		BC4.1	Verbal persuasion about capability 15.1	Sharing sessions/speed dating in MOPS sessions
	Intention	I2.4	Habit formation (routine exercise) 8.3	Health goal setting with a buddy
MOPS (organizational)	Knowledge	K5.1	Credible source 9.1	Leverage available networks
	Skills	S5.7	Action planning 1.4	MOPS sessions
		S5.8	Skill training (build skills for MOPS) 4.1	Peer coaching groups/interest groups
		S5.8	Social support (unspecified) 3.1	Peer coaching groups
		S5.8	Social support (unspecified) 3.1	Peer coaching groups
		S5.8	Social support (emotional) 3.3	Interest groups
		S5.8	Social support (emotional) 3.3	Interest groups
		S5.5	Goal setting (incorporate health promotion into activities) 1.1 Action planning 1.4	Mentor programs
	Social influences	SI5.3	Action planning 1.4	MOPS sessions
			Restructuring the physical environment 12.1	MOPS sessions
	Social/professional role and identity	SP5.4	Social support (practical) 3.2	Foster accountability and vulnerability
			Modelling of the behaviour 6.1	Foster accountability and vulnerability
		SP5.9	Skills training 4.1	Peer training modules
	Beliefs about capabilities (self‐efficacy)	BC5.2	Skills training 4.1	Peer training modules
	Beliefs about consequences (attitude)	BO5.4	Feedback on behaviour 2.2	Foster accountability and vulnerability
		BO5.6a	Feedback on behaviour 2.2	Foster accountability and vulnerability
		BO5.6b	Feedback on behaviour 2.2	Foster accountability and vulnerability

Abbreviations: BCT, behaviour change technique; MOPS, Mothers of Preschoolers.

^a^ Refer to Supporting Information S1: Table S4 for reference to the change objectives.

^b^ Constructs derived from literature.

^c^ Constructs derived from co‐design sessions.

The expert advisory group suggested learning the unique skills and prior experiences of mothers within the group such as cooking, pilates, yoga or physical activity of some sort and leverage on those skills to develop a sustainable program that fits conveniently into MOPS sessions. They suggested building a curriculum that incorporates the program ideas as components of one program and providing training to mothers to enable independent program delivery.

## 4 DISCUSSION

In this article, we describe the application of a participatory design methodology integrated with IM (steps 1–3) to inform the co‐design of program components that support mothers with young children (5 years and under) to reduce postpartum weight retention and improve health and wellbeing. To the best of our knowledge, this study is the first to incorporate evidence from reviews, stakeholder data, behaviour change theories and utilize a participatory approach to develop program components that can be delivered by mothers and integrated within existing services for mothers (i.e., mothers' group). We attempted to address the challenges of poor engagement and high attrition which are inherent in existing postpartum lifestyle intervention programs.^5^, ^34^ This study provides valuable insight for future researchers and program developers, addressing the current evidence gap in sufficient reach and engagement among mothers.^5^, ^6^, ^34^

The participatory design approach allowed us to design program components that were grounded in theory and evidence and to map strategies to mechanisms and methods that bring about the needed changes for mothers with young children.^17^ This methodology enabled us to consider and address several factors that influence behaviour at the individual and organizational levels. The qualitative component of this work supported the identification of resources and specific contextual needs of the mothers' group in supporting the health and wellbeing of mothers with young children. This facilitated the tailoring of the program components to their specific needs to promote better adherence.^35^

From the systematic reviews we undertook as part of the needs assessment and the focus group discussions, it was evident that social support was necessary for engaging in healthy lifestyle behaviours in postpartum women.^11^ This agrees with previous findings in postpartum lifestyle programs and highlights the importance of enlisting community groups and nontraditional health service providers to support healthy lifestyles.^36^, ^37^ Utilizing nonprofessional avenues for support is a potentially sustainable pathway for program delivery that reduces the burden on the limited time of health professionals. Mothers' groups are a support network for mothers with babies of similar ages and are a viable and cost‐effective method to provide community‐based support to mothers.^38^, ^39^ We found that mothers in this community group were positively disposed towards peer coaching and valued experienced mothers as mentors.^38^ Previous research has utilized peer support to promote weight control and the overall health and wellbeing of mothers through training and supporting peer mentors.^37^, ^40^ Peer‐led interventions offer a valuable low‐resource and sustainable strategy to provide necessary ongoing support to mothers with young children to reduce postpartum weight retention and mitigate the burden of maternal obesity.^41^ Training mothers to deliver programs may be an effective strategy that leverages their skills and experiences while simultaneously addressing their need for support.

Provision of practical support to relieve household chores including meal preparation, and childminding is critical to enabling women to engage in self‐care and health‐promoting behaviours early postpartum. This finding aligns with the current literature including our systematic review.^11^, ^36^ This is very important because many women prioritize their role as mothers and their family responsibilities over their self‐care.^36^, ^42^ Therefore, programs that address this need by providing relief from these responsibilities are likely to result in better engagement. The provision of childminding services during sessions was a major strength of the MOPS mothers' group. Delivering healthy meals to mothers who had recently given birth or were facing difficulties such as illnesses or loss was considered beneficial in supporting their health and wellbeing, offering an opportunity for mothers to mutually support one another during life challenges. This was particularly important for participants in this study given that 69% were migrants and had limited access to family support.

Another key finding of our focus group discussion is the need for inspiring leadership that values motherhood. This is necessary to achieve the program outcomes of increasing self‐esteem and self‐worth, coping with stress and supporting mothers to build confidence. The current health approaches do not do this, but rather employ a deficit‐based approach, which views mothers with postpartum weight retention as a ‘problem’. Our program components suggest a strength‐based approach, looking into the resources and strengths of mothers, elevating and valuing motherhood, and validating the successes of individual mothers within the group, thereby building confidence and self‐worth. One systematic review that formed part of our needs assessment revealed the need for increasing self‐worth and enjoyment of the activity to promote healthy lifestyles in postpartum women.^11^ Previous research also suggests that self‐worth is central to physical activity adherence.^43^ Therefore, having a program that builds confidence and self‐worth in mothers is likely to result in intrinsic motivation (a key component of behaviour change) to improve lifestyle behaviours. This might be the missing key to enabling this population group to be motivated; inspiring leadership sets the culture for it.

We also found that providing learning opportunities for mothers through health education and engaging experts was considered valuable by mothers. Health professional‐delivered interventions have been reported to be associated with effective weight loss in lifestyle interventions for postpartum women.^28^ This may be because health professionals are viewed as a source of credible information.^36^ However, long‐term lifestyle support is essential for weight management and health professionals have limited capacity to provide ongoing lifestyle support to postpartum women because of the time and resources required to take on this role.^21^ The recent coronavirus disease‐19 pandemic has put more strain on the healthcare system in many countries, including Australia, further reducing access to health services, particularly preventive services.^44^ Therefore, there is a need to consider alternative pathways and models of care for sustainable partnerships between health professionals and community service providers to provide credible, long‐term support for the ongoing provision of lifestyle counselling and support for postpartum women to address maternal obesity.

Expanding the reach and increasing accessibility to mothers who live farther away was considered necessary to increase the program's impact. Literature suggests that enlisting the support of community organizations can expand the reach and effectiveness of health promotion activities.^45^ Also, programs with good penetration and participation rates are those integrated with existing services used by postpartum women.^46^ Integrating programs into existing services reduces the barriers of time constraints and lack of childcare that usually deter mothers from engaging in lifestyle programs.^11^ Although our program components have been developed within the context of a mothers' group in Australia, the lessons learnt have relevance for researchers and health practitioners internationally for adaptation and translation in similar settings. Also, MOPS being an international group could extend the findings more broadly, and beyond Australia.

Our study has several strengths. It represents a major contribution to postpartum lifestyle research as it is the first, to our knowledge, to systematically develop evidence‐ and theory‐based peer‐led lifestyle program components that can be integrated within community mothers' groups. The program design was strengthened by the community participatory approach. Capturing the experiences and perspectives of the target population optimizes potential feasibility for adoption and acceptability. This enhances the translation of health research into practice in real‐world settings. Incorporating the IM approach guided the development of program components in partnership with key stakeholders and allowed us to explicitly incorporate theory and evidence in the development process.

Some limitations should be acknowledged. We did not involve maternal and child health (MCH) services, a potential stakeholder that has been identified as an acceptable avenue for program delivery by postpartum women.^47^ However, community mothers' groups provide more opportunities for social support and engagement for a longer duration than MCH services. Furthermore, although the family plays a significant role in influencing the lifestyle choices of mothers, we did not include influencing family members' behaviour as a program outcome.^48^ While our project resources did not enable expansion to the family, it is recommended that future research consider incorporating the family or the key support person as an interpersonal level of the program. In addition, further research is needed to develop the curriculum, training manuals and other materials, pilot, implement, and evaluate the program on effects and processes corresponding to steps 4, 5 and 6 of the IM approach. These findings are important for practice and policy to urgently address obesity issues in mothers through low‐resource programs that have the potential to increase reach and engagement through integration with existing services.

## 5 CONCLUSION

This article describes how we systematically applied a participatory design methodology combined with a modified IM approach to co‐design evidence‐informed and theory‐based program components, to reduce postpartum weight retention and improve the health and wellbeing of mothers with young children. In future research, the program curriculum and materials must be developed, piloted, implemented, and evaluated. Leveraging mothers' groups for the implementation of lifestyle programs to reduce postpartum weight retention and support the health and wellbeing of mothers has the potential to improve program reach and engagement, leading to large‐scale impact. A novel finding of our study is the focus on a strength‐based approach of valuing motherhood through inspiring leadership to improve self‐worth and confidence for healthy lifestyle behaviours.

## AUTHOR CONTRIBUTIONS

**Maureen Makama**: Conceptualization; methodology; software; validation; formal analysis; investigation; data curation; writing—original draft preparation; writing—review and editing; visualization; project administration. **Helen Skouteris**: Methodology; validation; investigation; writing—review and editing; visualization. **Lisa J. Moran**: Methodology; validation; investigation; writing—review and editing; visualization. **Briony Hill**: Methodology; validation; investigation; writing—review and editing; visualization. **Leanne M. Redman**: Methodology; validation; investigation; writing—review and editing; visualization. **Siew Lim**: Conceptualization; methodology; software; validation; formal analysis; investigation; resources; data curation; writing—review and editing; visualization; supervision; project administration; funding acquisition. All authors have read and agreed to the published version of the manuscript.

## CONFLICT OF INTEREST STATEMENT

The authors declare no conflict of interest.

## ETHICS STATEMENT

Ethics approval for this study was obtained from the Monash University Human Research Ethics Committee (HREC) (Project number: 31212) on 13 December 2021. Informed consent was provided by all participants before participating in the study.

## Supporting information

Supporting information.

## Data Availability

The data supporting this study's findings are not publicly available due to the potential for reidentification of participants but are available from the corresponding author on reasonable request.

## References

[1] World Health Organization . Obesity and Overweight. World Health Organization; 2016. https://www.who.int/news-room/fact-sheets/detail/obesity-and-overweight

[2] Gunderson EP . Childbearing and obesity in women: weight before, during, and after pregnancy. Obstet Gynecol Clin North Am. 2009;36(2):317‐332. 10.1016/j.ogc.2009.04.001 19501316 PMC2930888

[3] Gore SA , Brown DM , West DS . The role of postpartum weight retention in obesity among women: a review of the evidence. Ann Behav Med. 2003;26(2):149‐159. 10.1207/s15324796abm2602_07 14534032

[4] Endres LK , Straub H , McKinney C , et al. Postpartum weight retention risk factors and relationship to obesity at 1 year. Obstet Gynecol. 2015;125(1):144‐152. 25560116 10.1097/AOG.0000000000000565 PMC4286308

[5] McKinley MC , Allen‐Walker V , McGirr C , Rooney C , Woodside JV . Weight loss after pregnancy: challenges and opportunities. Nutr Res Rev. 2018;31(2):225‐238. 29984681 10.1017/S0954422418000070

[6] Makama M , Skouteris H , Moran LJ , Lim S . Reducing postpartum weight retention: a review of the implementation challenges of postpartum lifestyle interventions. J Clin Med. 2021;10(9):1891. 10.3390/jcm10091891 33925502 PMC8123857

[7] Farpour‐Lambert NJ , Ells LJ , Martinez de Tejada B , Scott C . Obesity and weight gain in pregnancy and postpartum: an evidence review of lifestyle interventions to inform maternal and child health policies. Front Endocrinol. 2018;9:546. 10.3389/fendo.2018.00546 PMC6168639 30319539

[8] Luke S , Kirby RS , Wright L . Postpartum weight retention and subsequent pregnancy outcomes. J Perinat Neonatal Nurs. 2016;34(4):292‐301. 26866522 10.1097/JPN.0000000000000160

[9] Wallace JM , Bhattacharya S , Campbell DM , Horgan GW . Inter‐pregnancy weight change and the risk of recurrent pregnancy complications. PLoS One. 2016;11(5):e0154812. 27145132 10.1371/journal.pone.0154812 PMC4856284

[10] Lim S , Hill B , Teede HJ , Moran LJ , O'Reilly S . An evaluation of the impact of lifestyle interventions on body weight in postpartum women: a systematic review and meta‐analysis. Obes Rev. 2020;21(4)e12990. 31914234 10.1111/obr.12990

[11] Makama M , Awoke MA , Skouteris H , Moran LJ , Lim S . Barriers and facilitators to a healthy lifestyle in postpartum women: a systematic review of qualitative and quantitative studies in postpartum women and healthcare providers. Obes Rev. 2021;22(4):e13167. 10.1111/obr.13167 33403746

[12] Skivington K , Matthews L , Simpson SA , et al. A new framework for developing and evaluating complex interventions: update of Medical Research Council guidance. BMJ. 2021;374:n2061. 10.1136/bmj.n2061 34593508 PMC8482308

[13] Haldane V , Chuah FLH , Srivastava A , et al. Community participation in health services development, implementation, and evaluation: a systematic review of empowerment, health, community, and process outcomes. PLoS One. 2019;14(5):e0216112. 10.1371/journal.pone.0216112 31075120 PMC6510456

[14] French SD , Green SE , O'Connor DA , et al. Developing theory‐informed behaviour change interventions to implement evidence into practice: a systematic approach using the Theoretical Domains Framework. Implement Sci. 2012;7:38. 10.1186/1748-5908-7-38 22531013 PMC3443064

[15] Michie S , Johnston M , Francis J , Hardeman W , Eccles M . From theory to intervention: mapping theoretically derived behavioural determinants to behaviour change techniques. Appl Psychol. 2008;57(4):660‐680.

[16] O'Cathain A , Croot L , Duncan E , et al. Guidance on how to develop complex interventions to improve health and healthcare. BMJ Open. 2019;9(8):e029954. 10.1136/bmjopen-2019-029954 PMC6701588 31420394

[17] Eldredge LKB , Markham CM , Ruiter RA , Fernández ME , Kok G , Parcel GS . Planning Health Promotion Programs: An Intervention Mapping Approach. Wiley; 2016.

[18] Fernandez ME , Ruiter RAC , Markham CM , Kok G . Intervention mapping: theory‐ and evidence‐based health promotion program planning: perspective and examples. Front Public Health. 2019;7:209. 10.3389/fpubh.2019.00209 31475126 PMC6702459

[19] Corbie‐Smith G , Akers A , Blumenthal C , et al. Intervention mapping as a participatory approach to developing an HIV prevention intervention in rural African American communities. AIDS Educ Prev. 2010;22(3):184‐202. 10.1521/aeap.2010.22.3.184 20528128 PMC3037273

[20] Anselma M , Altenburg TM , Emke H , et al. Co‐designing obesity prevention interventions together with children: intervention mapping meets youth‐led participatory action research. Int J Behav Nutr Phys Act. 2019;16(1):130. 10.1186/s12966-019-0891-5 31831006 PMC6909512

[21] Krishnamurti T , Simhan HN , Borrero S . Competing demands in postpartum care: a national survey of US providers' priorities and practice. BMC Health Serv Res. 2020;20:284. 32252757 10.1186/s12913-020-05144-2 PMC7137294

[22] MOPS International. Accessed August 28, 2022. https://www.mops.org/

[23] Centre of Perinatal Excellence COPE. https://www.cope.org.au/new-parents/first-weeks/community-resources-providing-support/

[24] MOPS Australia. Accessed August 28, 2022. https://mops.org.au/

[25] Michie S , Van Stralen MM , West R . The Behaviour Change Wheel: a new method for characterising and designing behaviour change interventions. Implement Sci. 2011;6(1):42. 21513547 10.1186/1748-5908-6-42 PMC3096582

[26] Atkins L , Francis J , Islam R , et al. A guide to using the Theoretical Domains Framework of behaviour change to investigate implementation problems. Implement Sci. 2017;12(1):77. 28637486 10.1186/s13012-017-0605-9 PMC5480145

[27] Cane J , O'Connor D , Michie S . Validation of the Theoretical Domains Framework for use in behaviour change and implementation research. Implement Sci. 2012;7(1):37. 22530986 10.1186/1748-5908-7-37 PMC3483008

[28] Lim S , Liang X , Hill B , Teede H , Moran LJ , O'Reilly S . A systematic review and meta‐analysis of intervention characteristics in postpartum weight management using the TIDieR framework: a summary of evidence to inform implementation. Obes Rev. 2019;20(7):1045‐1056. 30942550 10.1111/obr.12846

[29] Lim S , Hill B , Pirotta S , O'Reilly S , Moran L . What are the most effective behavioural strategies in changing postpartum women's physical activity and healthy eating behaviours? A systematic review and meta‐analysis. J Clin Med. 2020;9(1):237. 31963150 10.3390/jcm9010237 PMC7019954

[30] von Kodolitsch Y , Bernhardt A , Robinson P , et al. Analysis of strengths, weaknesses, opportunities, and threats as a tool for translating evidence into individualized medical strategies (I‐SWOT). Aorta. 2015;03(03):98‐107. 10.12945/j.aorta.2015.14.064 PMC4820345 27069939

[31] Braun V , Clarke V . Using thematic analysis in psychology. Qual Res Psychol. 2006;3(2):77‐101. 10.1191/1478088706qp063oa

[32] Michie S , Richardson M , Johnston M , et al. The behavior change technique taxonomy (v1) of 93 hierarchically clustered techniques: building an international consensus for the reporting of behavior change interventions. Ann Behav Med. 2013;46(1):81‐95. 23512568 10.1007/s12160-013-9486-6

[33] Michie S , Atkins L , West R . The Behaviour Change Wheel: A Guide to Designing Interventions. Silverback Publishing; 2014. https://ww.behaviourchangewheel.com

[34] Lim S , O'Reilly S , Behrens H , Skinner T , Ellis I , Dunbar JA . Effective strategies for weight loss in post‐partum women: a systematic review and meta‐analysis. Obes Rev. 2015;16(11):972‐987. 26313354 10.1111/obr.12312

[35] Beck C , McSweeney JC , Richards KC , Roberson PK , Tsai PF , Souder E . Challenges in tailored intervention research. Nurs Outlook. 2010;58(2):104‐110. 10.1016/j.outlook.2009.10.004 20362779 PMC3136169

[36] Dennison RA , Ward RJ , Griffin SJ , Usher‐Smith JA . Women's views on lifestyle changes to reduce the risk of developing type 2 diabetes after gestational diabetes: a systematic review, qualitative synthesis and recommendations for practice. Diabetic Med. 2019;36(6):702‐717. 30723968 10.1111/dme.13926 PMC6563496

[37] Small R , Taft AJ , Brown SJ . The power of social connection and support in improving health: lessons from social support interventions with childbearing women. BMC Public Health. 2011;11(5):S4. 10.1186/1471-2458-11-S5-S4 PMC3247027 22168441

[38] Law KH , Dimmock JA , Guelfi KJ , et al. A peer support intervention for first‐time mothers: feasibility and preliminary efficacy of the mummy buddy program. Women Birth. 2021;34(6):593‐605. 10.1016/j.wombi.2020.10.009 33160896

[39] Scott D , Brady S , Glynn P . New mother groups as a social network intervention: consumer and maternal and child health nurse perspectives. Aust J Adv Nurs. 2001;18(4):23‐29. 11878547

[40] Cahill AG , Haire‐Joshu D , Cade WT , et al. Weight control program and gestational weight gain in disadvantaged women with overweight orobesity: a randomized clinical trial. Obesity. 2018;26(3):485‐491. 10.1002/oby.22070 29464907 PMC5826625

[41] Lim S , Lee WK , Tan A , et al. Peer‐supported lifestyle interventions on body weight, energy intake, and physical activity in adults: a systematic review and meta‐analysis. Obes Rev. 2021;22(12):e13328. 10.1111/obr.13328 34387399

[42] Carter‐Edwards L , Østbye T , Bastian LA , Yarnall KS , Krause KM , Simmons TJ . Barriers to adopting a healthy lifestyle: insight from postpartum women. BMC Res Notes. 2009;2(1):161. 19686601 10.1186/1756-0500-2-161 PMC2746232

[43] Huberty JL , Ransdell LB , Sidman C , et al. Explaining long‐term exercise adherence in women who complete a structured exercise program. Res Q Exerc Sport. 2008;79(3):374‐384. 18816949 10.1080/02701367.2008.10599501

[44] Moynihan R , Sanders S , Michaleff ZA , et al. Impact of COVID‐19 pandemic on utilisation of healthcare services: a systematic review. BMJ Open. 2021;11(3):e045343. 10.1136/bmjopen-2020-045343 PMC7969768 33727273

[45] Stephens KK , Rimal RN , Flora JA . Expanding the reach of health campaigns: community organizations as meta‐channels for the dissemination of health information. J Health Commun. 2004;9(sup1):97‐111. 10.1080/10810730490271557 14960406

[46] Dasgupta K , Terkildsen Maindal H , Kragelund Nielsen K , O'Reilly S . Achieving penetration and participation in diabetes after pregnancy prevention interventions following gestational diabetes: a health promotion challenge. Diabetes Res Clin Pract. 2018;145:200‐213. 29684615 10.1016/j.diabres.2018.04.016

[47] Makama M , Chen M , Moran LJ , et al. Postpartum women's preferences for lifestyle intervention after childbirth: a multi‐methods study using the TIDieR Checklist. Nutrients. 2022;14(20):4229. 10.3390/nu14204229 36296913 PMC9611337

[48] Moran LJ , Lee J‐K , Jones D , Fronberg K , Feinberg ME . Coparenting‐focused preventive intervention reduces postnatal maternal BMI and buffers impact of cortisol. Obesity. 2022;30(8):1564‐1572. 10.1002/oby.23466 35854331 PMC9543348

