# Supplementary material for: Co‐designing a community lifestyle intervention program to reduce postpartum weight retention
Source: Health Expect. 2023 Nov 3;27(1):e13905. doi: 10.1111/hex.13905 (PMC10726270; doi:10.1111/hex.13905)
Supplement: Supplementary file 1 — Supporting information. [file HEX-27-e13905-s001.docx]

# **Online Resource for:**

# **Co-designing a community lifestyle intervention program to reduce postpartum weight retention**

Supplementary Table S1: Focus group discussion guide

| **Strengths:** |
| --- |
| 1) Could you tell me about anything MOPS is doing in terms of promoting health and wellbeing of mothers with young children? What strategies and approaches do you currently use to help support the health and wellbeing of your members? What worked well? What did not? |
| **Weaknesses:** |
| 2) What barriers do you foresee, for MOPS in supporting the health and wellbeing of your members? |
| **Opportunities:** |
| 3) If everything is possible, what would you like to see MOPS do to promote the health and wellbeing of mothers with young children? |
| 4) What might help you overcome these barriers (what resources, tools or support would be needed to help MOPS better support the health and wellbeing of mothers with young children)? |
| **Threats:** |
| 5) What might make it difficult (be a hindrance) for MOPS to take on promoting the health and wellbeing of mothers with young children. |

Supplementary Table S2. Thematic analysis: Facilitators and barriers faced by MOPS in improving the health of its members

| Initial codes | Quotes | Subthemes | Themes |
| --- | --- | --- | --- |
| Helps family relationships (supports healthy family relationship, healthy marriages) | even just the health of my relationships with my husband and my children to realize that this stage that we're in, some of the things I'm encountering is normal. - #4 | Building relationships between mothers and others in the community | Creating a supportive environment that enables peer and social support |
| Multigenerational community | They're the grandmother figure, the grandfather figure, which is wonderful, especially in our community where there are a lot of migrant moms connecting in with the group, and they don't have extended family living locally. - #12 |  |  |
| Encouragement | They're encouraging you to bring your kids and you know, the picture that they have of them working out. Um, just has toddlers everywhere and I just go, "Oh, okay. Well, that makes it so much more welcoming because I know my two will climb all over me." Um, but so will everyone else's, and I think it just makes it more comfortable because you know, that that's accepted and it's okay. #13 | Supportive environment |  |
| Community support | And so I keep coming back to a program such as this because I think it provides beautiful community encouragement and support from others. I think it's good for mothers to see that they're not going through the journey alone. -#8 |  |  |
| Mutual respect and acceptance | And, uh, is-is very healthy environment for a mother. And I find out they open my mind and they respect me the way I am, and they connect me with the community straight away, and is changed me.- #10 | Peer support |  |
| Similar struggles | Um, and so just hearing other women and other moms share about their struggles. I started to realize like, "Oh, I'm not the only one going through this particular struggle. - #4 |  |  |
| Looking for company | looking for like company and-and other moms that are at home that can share their time with me – #5 | Provides company |  |
| Social connection and love | Like when I- when I finally made it to MOPS, it was almost just like a-a breath of fresh air just over me because, um, if I didn't, you know, I-I missed the connection and it just feeds my soul. - #7 |  |  |
| Free meals for mothers experiencing hardships | free meals are funded to reach out to bless those moms who, um, you know, who just got a recent diagnosis that their child is, um, experiencing something just so shocking. - #12 | Practical support | Providing practical support such as delivering meals |
| Meal sharing | I think even just offering to bring over a meal and, um, a cup of tea or to look after children. – #13  …our group is really good at doing is to provide meals for each other and during lockdown, we did, um, like food hug deliveries. - #12 |  |  |
| Reciprocal support | So like if I need a babysitter, for instance, I could say, "Hey," at anybody, "Can I drop my kids ..." And then … I can reciprocate that, um, you know, take someone else's kids. Um, and so the support can continue, even outside of the actual meeting. - #4 |  |  |
| Practical support | when I had COVID, um, cause my family is far, all my friends are far…the MOPS community was the one who came and, you know, dropped off medication and just, yeah, just helped me because they were local. So that's been really, really helpful. - #6 |  |  |
| Mothers’ resort/hotel/restaurant | the other aspect of-of having a built-in restaurant is that moms who aren't staying in this hotel, um, they could order these nutritious- these nutritious meals that have been specially curated, right, for the wellbeing and the nurturing of moms as they're breastfeeding their kids, or, um, whatever part of their pregnancy journey, um, they're on perhaps. - #12 | Mothers’ resort/hotel/restaurant |  |
| Value in mothers | just the fact that…you know, that you're not alone. You know that you're not such a failure as a mom, you know, it just gave me so much confidence and I've only attended one session…I pulled myself out from, and now I'm-I'm seriously eating better. And, you know, I just-- it just gave me so much confidence and I'm eating better... I actually watch what I wear, you know, I actually like try to look good because, and feel good because, yeah, because I guess I was uplifted.- #11 | Values motherhood | Inspiring leadership that values motherhood |
| Nurture mothers | sometimes you even lose a bit of yourself because you're like, "Where's my identity now?" … I've left a career to become a mother,… so I just love how MOPS champions mothers. It gives opportunity for them to have their souls fed…And then the mother's are nurtured as well. -#8 |  |  |
| MOPS champions mothers | MOPS is so intentional to sow into the mom's life and to build relationships between the moms and just there's so much joy. So I think that that is so good for our health. - #8 |  |  |
| Inspiring leadership | being introduced to MOPS by the actual CEO of MOPS International. She, like, flew down from America and, like, gave a talk about-- Um, like, a really inspiring talk. - #2 |  |  |
| Goal to help mother flourish | love radically because MOPS was birthed from this desire to want to, um, not only cherish and nourish mothers, so that they may flourish. – #2 |  |  |
| Mentors | Um, from there, uh, we have mentors as well. So moms who have raised their children past the preschooler age that can speak into, um, and give hope and encouragement to moms who are still in-in that, um, stage of parenting preschoolers. - #12 | Mentoring from older mums |  |
| Freely talk about beliefs | I think too, that's also what makes MOPS such a unique place because you can freely talk about, um, your beliefs as well and-and have that Christian support network too, um, or extend that pastoral care. - #13 | Pastoral care |  |
| Mental health education | but also I would love to see education for, uh, the mental health as well..hard for us to admit that we might be struggling in one area or another, but, um, with two of my three children, I had-- I'm pretty sure I was struggling with undiagnosed postpartum depression. - #4 | Focuses on mother’s mental health and wellbeing | Provides learning opportunities that focus on mothers’ health and well-being |
| Physical health education | That was the biggest thing. Um, and an understanding like how foods could give me an insulin spike and enable me to gain more weight. So, um, yeah, so physical health has always been in the forefront of my mind, but I've never- I've never engaged in a community, uh, group to focus on that part of my wellbeing. –#1 | Focus on physical health |  |
| Engage experts | local dentist or local pediatrician, um, experts, uh, we've had, uh, authors come in as well, um, who might share about, um, one really powerful session was about motherly guilt and um, yeah, so just thinking about the theme and then reaching out for community support. Sometimes also, if there are mothers within our group who have expertise or work experience in a particular field, we get them to share as well. -#12 | Engage experts |  |
| Group discussion | We discuss different questions along that theme that we had for that day…group discussion time has been particularly enriching for me - #4 | Health education |  |
| By mums for mums | what makes MOPS unique and special is that it's moms helping moms that there's a-a limit to what moms can actually do, because you have your own kids who are pre-schoolers. Um, and you may also be juggling work with all of that so you're really time for, in trying to give to others and-and trying to extend it and grow it. - #13 |  |  |
| Nutrition education | The first and foremost thing would have to be education. Um, it's fair enough that, um, like we all need to eat to live, but choosing the right foods for us is something that has to be told to us by a professional. - #1 |  |  |
| Pelvic floor education | I think, too, adding to that pelvic floor education too. Um, that's something I've struggled with quite a bit and nobody told me. I didn't end up working on my pelvic floor until I almost got pregnant with my third child and, um, so I thought, you know, am I just broken - #4 |  |  |
| Organizations for support | it's who can you get on board to help with childcare or to make those connections with other organizations, um, or to approach other, you know, to make those applications for grant funding and for all that type of thing - #3 | Partnership with other organisations to promote the health of mothers | Reach and accessibility of MOPS |
| Accessible at all times | it was really good to have online as well, because sometimes you just-- it doesn't fit your schedule, or, um, you just can't make it or the kids are sick … So yeah, I really enjoyed the online gatherings actually. - #9 | Expand reach and access |  |
| Far reach | people who are further out, it would be lovely to have those little communities spring up. - #4 |  |  |
| Having online sessions is beneficial | … last year doing lockdown, MOPS was… online, um, and I could join because, um, you know, all my kids are asleep. And it was like, just to catch up with other mums and there was heaps of support from the MOPS group….And, um, it was just really good for my mental health, because, um, it was really hard time. - #9 |  |  |
| Expansion and availability | So I-I would just love to see it expand and grow and be available, um, at more times than just what it is. - #13 | Time and place barriers |  |
| Funding options | o I think, um, you know, it's who can you get on board to help with childcare or to make those connections with other organizations, um, or to approach other, you know, to make those applications for grant funding and for all that type of thing. -#13 | Limited resources |  |
| Resource barrier | I think too, because it's also affiliated with a religious, um, organization that a lot of grants, um, you know, there's the local council grant, but we're not eligible for that because it's affiliated with the church. - #13 |  |  |
| Carers | I talked to MOPS about a lot of friends that I come across in the community, but I realize, um, I have to be mindful of the capacity of the carers and-and, you know, space wise because we don't want to, um, over saturate the-the carers space with children - #7 | Limited childminders | Lack of resources for childminding service |

Supplementary Table S3. Key findings from the literature reviews

| Review paper | Description | Key findings |
| --- | --- | --- |
| Lim et al 2019[1] | Systematic review and meta-analysis of 33 studies that evaluated the intervention characteristics associated with weight loss in postpartum women (within 2 years of delivery) using the Template for Intervention Description and Replication (TIDieR) framework. | - Combined diet and physical activity interventions have greater effect on weight loss than physical activity only - Health professional-delivered interventions have greater weight loss than interventions delivered by non-health professional - The extent of weight loss was not influenced by intervention intensity, duration or setting |
| Lim et al 2020[2] | Systematic review and meta-analysis of 46 studies that included 3905 women to describe the associations between behavioural strategies and change in weight, diet, and physical activity in postpartum women. | - Postpartum lifestyle interventions significantly improved weight (mean difference -2.46 kg, 95% CI -3.65 to -1.27) and physical activity (standardised mean difference 0.61, 95% CI 0.20 to 1.02) but not energy intake. - No individual strategy was significantly associated with weight and physical activity outcomes. - Strategies such as problem solving, goal setting of outcome, reviewing outcome goal, feedback on behaviour, self-monitoring of behaviour, behavioural substitution and credible source were associated with greater reduction in energy intake. |
| Makama et al 2021[3] | Systematic review of 28 qualitative and quantitative studies to describe the barriers and facilitators to a healthy lifestyle in postpartum women (within 2 years of delivery) from the perspective of women and healthcare providers mapped to the Theoretical Domains Framework and the Capability, Opportunity, Motivation and Behaviour model. | Themes relating to barriers and facilitators identified according to COM-B model are:   - Capability: lack of knowledge regarding benefits of lifestyle behaviours, fatigue, physical and psychological health issues. - Opportunity: social support from partners, family, friends and health professionals, time availability and competing priorities, childcare needs and finances and the physical environment. - Motivation: self-worth, perception of ability and willingness to engage in lifestyle management, cultural and religious beliefs, identifying benefits of exercise and perception of personal health, enjoyment of the activity or food. |
| Makama et al 2021[4] | Narrative review of key determinants of effective implementation of postpartum lifestyle interventions using the Consolidated Framework for Implementation Research. | - Studies with good penetration and participation rates were conducted within existing health services for postpartum women or involved recruitment during pregnancy. - Barriers experienced by postpartum women lead to low levels of engagement in lifestyle interventions. - Health professionals face barriers of limited time and skills to provide support to postpartum women |

Supplementary Table S4. Matrices of change objectives for mothers of young children (individual level) and MOPS (organisational level) mapped to determinants based on the Theoretical Domains Framework.

| Performance objectives (PO) | Knowledge | Skills | Behavioural regulation | Social influences | Social/ professional role and identity | Beliefs about capabilities (self-efficacy) | Intention | Beliefs about consequences (attitude) |
| --- | --- | --- | --- | --- | --- | --- | --- | --- |
| PO 1.1 Eat less sugar and refined carbohydrates and more healthy fats | K1.1 Know how to read food labels | S1.1 Demonstrate an ability to read food labels and choose healthy options | BR1.1 Choose healthy food options |  |  |  |  |  |
| PO 1.2 Not eat children’s leftovers | K1.2 Acknowledge the need for portion control and mindful eating | S1.2 Practice portion control and mindful eating | BR1.2 Choose not to eat children’s leftovers |  |  | BC1.2 Express confidence in ability to avoid eating children’s leftovers |  |  |
| PO 1.3 Be conscious of the food they eat | K1.3 Acknowledge the need for portion control and mindful eating | S1.3 Practice portion control and mindful eating |  |  |  |  |  |  |
| PO 2.1 Have a consistent routine for newborn and self |  |  |  |  |  | BC2.1 Express confidence in ability to maintain a consistent routine |  |  |
| PO 2.2 Use deep breathing exercises |  | S2.2 Demonstrate ability to do deep breathing exercises |  |  |  |  |  |  |
| PO 2.3 Understand that even small amounts of exercise each day is okay e.g., 7 mins a day, 1 min per time | K2.3 List the benefits of small bouts of exercise |  |  |  |  |  |  |  |
| PO 2.4 Incorporate exercise into usual routine e.g., house cleaning, playdates |  | S2.4 Demonstrate ability to incorporate exercise into daily routine |  |  |  | BC2.4 Express confidence in ability to incorporate physical activity into daily schedules | I2.4 Make conscious decision to exercise daily |  |
| PO 2.5 Have someone to be accountable to e.g., partner or buddy |  |  |  | SI2.5 Enlist the support of a partner or buddy for accountability |  |  |  |  |
| PO 3.1 Value and prioritise themselves |  |  |  |  |  | BC3.1 Express confidence in ability to prioritise and value self |  |  |
| PO 3.2 Be willing to share struggles with someone |  |  |  |  |  | BC3.2 Express confidence in ability to share struggles with someone |  |  |
| PO 4.1 Learn strategies to cope with stress e.g., allocate a 1-hour stress time per day |  | S4.1 Have the skills to cope with stress |  |  |  | BC4.1 Express confidence in ability to manage stress |  |  |
| PO 5.1 Partner with local health/allied health professionals to provide education/training opportunities to mothers | K5.1 Describe how to identify partnership opportunities |  |  |  |  |  |  |  |
| PO 5.2 Refer mothers to professionals for help as needed |  |  |  |  |  | BC5.2 Express confidence in ability to refer mothers to professionals |  |  |
| PO 5.3 Create a safe space for mothers to be vulnerable |  |  |  | SI5.3 Create a safe environment for mothers |  |  |  |  |
| PO 5.4 MOPS leaders model vulnerability as a virtue |  |  |  |  | SP5.4 Express willingness to be vulnerable |  |  | BO5.4 Expect that modelling vulnerability will encourage mothers to open up |
| PO 5.5 Be intentional about pastoral care |  | S5.5 Demonstrate ability to provide pastoral care |  |  |  |  |  |  |
| PO 5.6 Be intentional in championing mothers, serving them in the way they need and making them feel known and loved |  |  |  |  |  |  |  | BO5.6.1 Expect that championing mothers will make them feel known and loved  BO5.6.2 Expect that championing mothers will help build their self-esteem/self-worth |
| PO 5.7 Incorporate exercise, dance and music, healthy eating, and health promotion into MOPS structure/activities/discussions |  | S5.7 Demonstrate ability to incorporate health promoting into MOPS activities |  |  |  |  |  |  |
| PO 5.8 Create interest groups for mothers e.g., meal planning, diet, weight loss |  | S5.8 Demonstrate ability to create interest groups for mothers |  |  |  |  |  |  |
| PO 5.9 MOPS leaders take up training opportunities to equip them to better support mothers e.g., by PANDA |  |  |  |  | SP5.9 Express willingness to be trained |  |  |  |

**References**

1. Lim S, Liang X, Hill B, Teede H, Moran LJ, O'Reilly S. A systematic review and meta‐analysis of intervention characteristics in postpartum weight management using the TIDieR framework: A summary of evidence to inform implementation. *Obesity Reviews*. 2019;20(7):1045-56.

2. Lim S, Hill B, Pirotta S, O’Reilly S, Moran L. What Are the Most Effective Behavioural Strategies in Changing Postpartum Women’s Physical Activity and Healthy Eating Behaviours? A Systematic Review and Meta-Analysis. *Journal of clinical medicine*. 2020;9(1):237.

3. Makama M, Awoke MA, Skouteris H, Moran LJ, Lim S. Barriers and facilitators to a healthy lifestyle in postpartum women: A systematic review of qualitative and quantitative studies in postpartum women and healthcare providers. *Obes Rev*. 2021;22(4):e13167;10.1111/obr.13167.

4. Makama M, Skouteris H, Moran LJ, Lim S. Reducing Postpartum Weight Retention: A Review of the Implementation Challenges of Postpartum Lifestyle Interventions. *J Clin Med*. 2021;10(9);10.3390/jcm10091891.
